# Aging and Mesenchymal Stem Cells: Basic Concepts, Challenges and Strategies

**DOI:** 10.3390/biology11111678

**Published:** 2022-11-18

**Authors:** Maria Fraile, Noemi Eiro, Luis A. Costa, Arancha Martín, Francisco J. Vizoso

**Affiliations:** 1 Research Unit, Fundación Hospital de Jove, Avda. Eduardo Castro, 161, 33920 Gijon, Spain; 2 Department of Emergency, Hospital Universitario de Cabueñes, Los Prados, 395, 33394 Gijon, Spain; 3 Department of Surgery, Fundación Hospital de Jove, Avda. Eduardo Castro, 161, 33920 Gijon, Spain

**Keywords:** mesenchymal stem cells, tissue homeostasis, aging, secretome, conditioned medium, extracellular vesicles, exosomes

## Abstract

**Simple Summary:**

Aging and frailty are complex processes implicating multifactorial mechanisms, which demand for new therapeutic strategies against their devastating effects. Mesenchymal stem cells (MSC) participate in a “galaxy” of tissue signals (proliferative, anti-inflammatory, and antioxidative stress, and proangiogenic, antitumor, antifibrotic, and antimicrobial effects) contributing to tissue homeostasis. However, MSC are also not immune to aging. Three strategies based on MSC have been proposed: remove, rejuvenate, or replace the senescent MSC. We propose the new strategy of “Exogenous Restitution of Intercellular Signalling of Stem Cells” (ERISSC). This concept is based on that the potential use of secretome from MSC, which are composed of molecules such as growth factors, cytokines, and extracellular vesicles and have the same biological effects as their parent cells. Therefore, this strategy allows us to avoid the drawbacks of cell therapy.

**Abstract:**

Aging and frailty are complex processes implicating multifactorial mechanisms, such as replicative senescence, oxidative stress, mitochondrial dysfunction, or autophagy disorder. All of these mechanisms drive dramatic changes in the tissue environment, such as senescence-associated secretory phenotype factors and inflamm-aging. Thus, there is a demand for new therapeutic strategies against the devastating effects of the aging and associated diseases. Mesenchymal stem cells (MSC) participate in a “galaxy” of tissue signals (proliferative, anti-inflammatory, and antioxidative stress, and proangiogenic, antitumor, antifibrotic, and antimicrobial effects) contributing to tissue homeostasis. However, MSC are also not immune to aging. Three strategies based on MSC have been proposed: remove, rejuvenate, or replace the senescent MSC. These strategies include the use of senolytic drugs, antioxidant agents and genetic engineering, or transplantation of younger MSC. Nevertheless, these strategies may have the drawback of the adverse effects of prolonged use of the different drugs used or, where appropriate, those of cell therapy. In this review, we propose the new strategy of “Exogenous Restitution of Intercellular Signalling of Stem Cells” (ERISSC). This concept is based on the potential use of secretome from MSC, which are composed of molecules such as growth factors, cytokines, and extracellular vesicles and have the same biological effects as their parent cells. To face this cell-free regenerative therapy challenge, we have to clarify key strategy aspects, such as establishing tools that allow us a more precise diagnosis of aging frailty in order to identify the therapeutic requirements adapted to each case, identify the ideal type of MSC in the context of the functional heterogeneity of these cellular populations, to optimize the mass production and standardization of the primary materials (cells) and their secretome-derived products, to establish the appropriate methods to validate the anti-aging effects and to determine the most appropriate route of administration for each case.

## 1. Introduction

The number of people older than 64 years will reach over 1.5 billion between 2019 and 2050, which means that the population of that age group will double compared to the current one [1]. In other words, within 30 years one in six people will be considered an “older adult” in the world [1]. The aging is characterized by physiological vulnerability due to both an inadequate response to stress and homeostasis balance. Therefore, at age 65 there is an elevated risk of neurodegenerative diseases, cardiovascular diseases, diabetes, cancers, osteoporosis, and osteoarthritis, among others [2,3]. In addition, elderly people are not only more frequently subjected to polypharmacy, but they are also more susceptible to its interactions and adverse effects in general [4]. For all of this, aging is increasing burden on our healthcare, particularly in countries with the highest longevity [5]. In fact, despite the accredited modern social and medical advances, the biological age did not grow in an equivalent dimension. On the contrary, the increasing life expectancy tends to be accompanied with increasing morbidity.

All of this causes the demand for new therapeutic alternatives in a growing part of society that, endowed with economic resources and accessibility to medical–scientific information, are raising the expectations of a better quality of life in the prelude to a vital chronology truncated by an exhausted physiopathology. In this scenario, the emergence of the paradigm of regenerative medicine can unleash a “perfect storm” of ideas and new approaches in order to face the challenge of a healthier longevity. Therefore, novel and urgent therapeutic strategies are necessary to help with age-related disorders by developing novel strategies of treatment.

Here, we will address the potential interest of MSC (mesenchymal stem cells) for their regulatory role in tissue homeostasis, their alterations in aging and associated diseases, and their possible value as the basis for a new therapeutic strategy.

## 2. Mechanisms of Aging

Aging is a complex process not only from the psychological and social point of view, but also from the biological context [6,7]. In fact, none of the existing definitions of aging fit the enormous complexity of the process [8,9].

**Figure 1:**
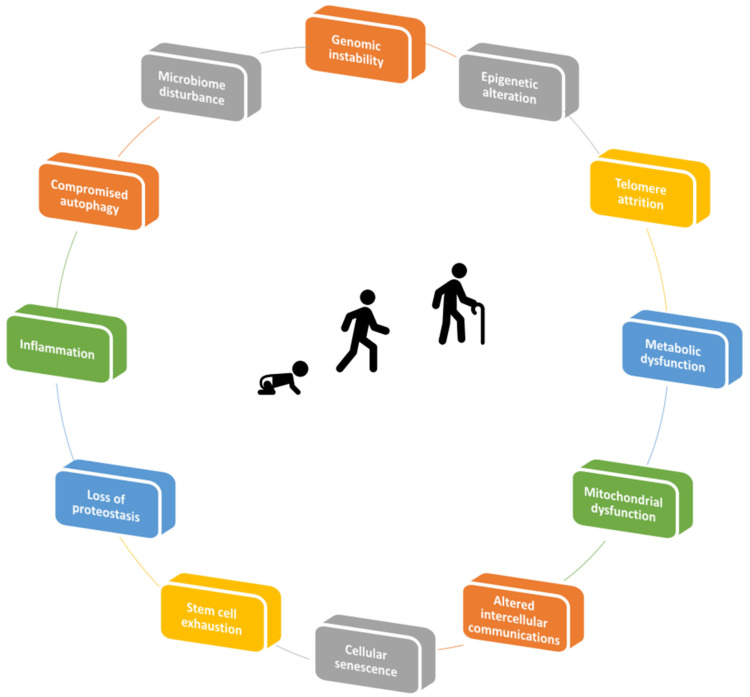
Hallmarks of aging.

There are several hallmarks which have characterized aging [10]. These include genomic instability, epigenetic alteration, telomere attrition, metabolic dysfunction, loss of proteostasis, mitochondrial dysfunction, stem cell exhaustion, cellular senescence, and altered intercellular communications (for review, see [11,12]). Tentatively, a narrative of the process that leads to aging can be constructed, starting from a concept that is the consequence of continuous endogens and/or exogenous stresses, including oxidative stress, replicative exhaustion, chemicals, or irradiation. These pernicious conditions affect adult somatic cells, inducing cell cycle arrest as well as resistance to cell death by necrosis, autophagy, or apoptosis. All of this collectively may induce a state of replicative senescence [13] (Figure 1).

When DNA replicates, the telomere is shortened. Continuous telomere erosion may drive in an irreversibly restricted proliferation, named replicative senescence. This process is intimately connected to oncogene-induced senescence. Activation and/or overexpression of oncogenes, such as RAF, MEK, cyclin E, and BRAF, not only drives senescence but is also a necessary step in tumorigenesis. Stress-induced premature senescence may be because of several stimulations, such as mechanical stress, hypoxia, osmotic stress, high glucose concentrations, ionizing radiation, heat shock, and reactive oxygen species (ROS) [14,15]. With regard to this latter stimulation, the dysregulation of the production of ROS and antioxidants causes both DNA and protein damage, as well as mitochondrial dysfunction, which impacts aging [16]. This is due to mitochondria, as cellular energy places contribute to key cell activities (apoptosis, initiation of signal transduction pathways, or cell matrix metabolism).

Another mechanism that has been associated with senescence is autophagy. This physiological process is necessary for maintaining cellular homeostasis. Autophagy prevents age-related cellular injury by removing cell damage components, such as proteins and mitochondria [17]. However, autophagy efficiency gradually decreases with the growth of age [18].

Epigenic modifications in histone regulation, which lead to silencing genes, are also strongly related to cellular senescence [19,20,21].

All of these senescent processes lead to morphological cellular changes characterized by flattened morphology with stress fibers, enlarged nuclei, and vacuolization due to the accumulation of macromolecules. Most of these macromolecules, such as the acidic senescence-associated β-galactosidase (SA-βGal), senescence-associated lysosomal α-L-fucosidase (SA-α-Fuc) [16], cell cycle regulators (p16INK4a, p21, p27, p53, pRB), and senescence-associated secretory phenotype (SASP) factors, are used as senescence markers. The SASP comprises growth factors (vascular endothelial growth factor (VEGF), hepatocyte growth factor (HGF), basic fibroblast growth factor (bFGF), insulin growth factor-1 (IGF-1), TGFβ), cytokines (interleukin-1β (IL-1β), IL-6), chemokines (monocyte chemoattractant protein-1 (MCP-1), IL-8), and extracellular proteases (matrix metalloprotease-1 (MMP-1), -3, -13). It is relevant to mention that SASP, by producing a self-perpetuating intracellular signaling loop in senescent cells, contributes to subclinical inflammation [22].

### 2.1. The Importance of the Relationship between Aging and Immune System

In general, the above-mentioned mechanisms associated with senescence, such as the incessant oxidative stress, telomere shortening, impaired autophagy, or epigenetic alterations, impact both the innate and adaptative immune systems (reviewed by [23]). All of this has led to a line of evidence between the immune system and aging, which can be narrowed down to two categories, namely immunosenescence and inflamm-aging [24]. The term immunosenescence refers to decline in the immune system competence with aging. With aging comes upregulated expression of inflammatory pathway genes in the monocytes/macrophages [25]; increased propensity to viral, bacterial and parasitic infection; cytokines such as TGF-β and IL-10 suppressing dendritic cells (DC) function and promoting the M2-type macrophage polarization; and the neutrophils decreasing their phagocytic ability and diminished bactericidal activity [26]. With regard to the natural killer (NK), it was reported that their antiviral capacity also decreases with age [27]. On the other hand, the toll-like receptors’ (TLRs) activity also declines with age [28,29].

It is relevant that senescent lymphocytes have a limited capacity in eliminating novel antigens, display a proinflammatory cytokine profile, can evade apoptosis, and favor the development of autoimmunity [30,31,32]. This incessant replication of T cells affects their proliferation capacity and leads to an immune cell refractory state [33,34]. Especially, B cells in older adults decrease their response capability toward pathogens [32], particularly in their switched memory B cells [31,34].

All of these proinflammatory changes associated with immunosenescence may drive to state of inflamm-aging, which, as proposed by the Prof. Claudio Franceschi in 2000, is an evolutionary perspective on immunosenescence [35]. Thus, inflamm-aging, which arises without a clear external stimulus, translates into a negative imbalance between innate and adaptive immunity.

## 3. Aging and Disease

Immunosenescence and inflamm-aging have been suggested to be largely responsible for the origin of the diseases of the elderly, such as chronic inflammatory diseases, autoimmune disorders, infections, and cancer. In an extreme stage, it may occur as a complex phenomenon named frailty, which has been defined as a generic condition of reduced strength, stamina, stability, and process and is a potential risk of morbidity and mortality [36].

It is relevant that most age-related diseases share a similar inflammatory pathogenesis. In addition, inflamm-aging may exacerbate these disease processes and their morbidity. This common inflammatory disease includes atherosclerosis, hypertension and myocardial infarction, Alzheimer’s disease, Parkinson’s disease, rheumatoid arthritis, and type II diabetes [22,37,38]. As a result of degenerating immunity, older age groups are also more susceptible to severe infections such as community-acquired pneumonia [39,40]. In addition, there are strong data pointing to higher morbidity from infections in the elderly population, reflected, for example, in developing cognitive decline post-infection [41]. It is remarkable that older adults have a higher autoimmunity, which is characterized by high levels of circulating T-regulatory cells (Treg) and a reduced CD4/CD8 ratio, which results in a predisposing imbalance to infection and cancer [42]. On the other hand, due to immune system dysfunction with aging, vaccines are less effective in the elderly population [43,44,45,46].

## 4. MSC and Tissue Homeostasis

MSC are defined by their capacity to adhere to plastic, by expressing a set of phenotypic markers (CD73+, CD90+, CD105+, CD11b– or CD14–, CD19– or CD79a–, CD34–, CD45–, HLA-DR–) and by their ability to differentiate toward osteoblasts, adipocytes, and chondrocytes [47]. Multiple effects of MSC, depending on their origin and/or heterogeneity, suggest a key role in controlling tissue homeostasis, which is truncated in several age-related diseases [48]. This may be in part because their mesodermal origin, whose biology leads to the constitution of a stroma intimately interconnected with both ectoderm- or endoderm-derived tissues. Other examples of specialized interstitial cells of mesoderm origin are interstitial cells of Cajal or the synovial fibroblasts [49,50]. With regard to MSC, there is growing evidence that they participate in a “galaxy” of intercellular signals contributing to tissue homeostasis [51,52]. In fact, a decrease or dysfunction of MSC have been related with inflammatory and degenerative base diseases such as diabetes mellitus (DM), rheumatoid arthritis (RA), or systemic lupus erythematosus (SLE) [53]. For all of these reasons, mesenchymal (stromal) stem cells are probably the adequate term. Due to their low immunogenicity, MSC have been safely transplanted autologously or allogeneically [48,54]. In the treatment of various diseases, such as graft-vs.-host disease (GVHD) [55], Crohn’s disease (CD) [56], diabetes mellitus (DM) [57], multiple sclerosis (MS) [58], and myocardial infarction (MI) [59], etc., today it is essential that MSC can exert their functions through a paracrine manner mainly by secretion of soluble factors (growth factors and cytokines), but also by the extracellular compartment. There are two main subtypes of extracellular vesicles (EVs): exosomes and microvesicles [60]. Exosomes, of less than 120 nm in size, are originated from the endosomal compartment. Microvesicles, which are between 100 and 500 nm, are formed by the budding of the plasma membrane. Apoptotic bodies are a type of EV (500–5000 nm) and are released upon the fragmentation of apoptotic cells. The content of EVs consist of nucleic acids (mRNA, DNA, or microRNAs), proteins, and lipids. EVs isolated from MSC have similar functions of the parental cell [60]. Globally, MSC-derived secretory products have anti-inflammatory, regenerative, and antioxidative stress, and antitumor, antifibrotic, and antimicrobial effects, which may positively impact aging frailty and aged-related diseases.

### 4.1. Anti-Inflammatory Effects

It is considered that MSC act on the immune system mainly by a paracrine manner rather than direct cell-to-cell contact [61,62]. MSC secrete soluble factors, which produce an immunomodulatory effect, such as TGF-β, hepatocyte growth factor (HGF), prostaglandin E2 (PGE2), indoleamine 2,3-dioxygenase (IDO), nitric oxide (NO), interferon-gamma (IFN-γ), IL-2, and IL-10. Consequently, MSC result in immunoregulatory effects on all of the immune cell types: (i) inhibit the proliferation of T cells [63] and attenuate their functionality [64,65]; (ii) influence B cells, reduce their plasmablast formation and induce regulatory B cells (Bregs) [66]; (iii) polarize monocytes (M0) toward the anti-inflammatory M2 phenotype [67] and reprogram M1 macrophages to the M2 phenotype [68]; (iv) suppress the proliferation of NK and their cytotoxicity; and (v) inhibit the differentiation and migration of dendritic cells (DCs) [69].

### 4.2. Regenerative Effects

MSC produce many active factors which contribute to tissue regeneration [70]. These factors include ECM proteins (MMP-1, -2, -3, -7; TIPM-1 and 2, elastin, collagens, laminin, and ICAM), growth factors (TGF-β, HGF, EGF, KGF, FGF, VEGF, PDGF, GF-1, NGF-3, BNDF, I G-CSF, or GM-CSF), and inflammatory proteins (MCP-1, PGE2, IL-1, -6, -8–11, -13). Another mechanism of regenerative effect is angiogenesis. MSC secreting molecular factors stimulate the proliferation and migration of endothelial cells. These factors include VEGF, ANG-1 y 2, PDGF, TGF-β1, TGF-α, EGF, FGF, CXCL5, MCP-1, and MMPs [71,72]. However, the secretion of these factors is reduced in aged MSC. On the contrary, aged MSC produce high levels of antiangiogenic factors (thrombospondin-1 (TBS1) and plasminogen activator inhibitor-1 (PAI-1)) [73,74]. On the other hand, there are data supporting the idea that MSC could promote neurogenesis and improve the neurological state in several studies, in vivo and in vitro [75,76].

### 4.3. Antioxidative Stress Effects

MSC express antioxidant enzymes, such as the antioxidant glutathione (GSH), glutathione peroxidase (Gpx), SOD1, SOD2, and catalase (CAT) [77], as well sirtuin (SIRT) and heat-shock protein 70 (HSP70) [78]. Consequently, in vitro studies have demonstrated the antioxidant effect of MSC on immune cells, fibroblasts, skeletal muscle cells, endothelial cells, cardiomyocytes, renal cells, hepatocytes, pancreatic islet cells, glial cells, and neurons. In addition, this protective effect of MSC was also proved by in vivo studies on aging, ischemic injuries, diabetes, gastrointestinal inflammation, infections, and radiation- or chemotherapy-induced cognitive disorders. MSC have antioxidant effects by the scavenging of free radicals via reactive oxygen species suppression, promoting endogenous antioxidant defenses and altering mitochondrial bioenergetics [79,80].

### 4.4. Antitumor Effects

Aging is a recognized condition that is highly linked to cancer development. If fact, the incidence of cancer is over 10-fold higher in the population over 65 years old [81]. It has been estimated that, especially, 40% of individuals aged over 65 years will have cancers [82]. In addition, it is known that the adverse effects of anticancer therapies increased with aging [83].

Several studies suggest that the pro or antitumor effect of MSC is dependent on their tissular origin and the type of tumor [84,85]. MSC originated in the uterus could become good candidates for cancer therapies [86].

MSC secrete high amounts of cytokines, which induce the inhibition of tumor growth in vivo in breast cancer cells such as IL12, IFN-α, CXCL10, LAP, DKK-1/3, TRAIL, TNFSF14 (also known as LIGHT), and FLT-3 ligand [87,88,89]. The antitumor effect of MSC may be also partly related to TIMP-1 and TIMP-2, present in their secretomes [88,90]. This might be due to the inhibition of MMPs, which is related with the loss of invasiveness of cancer cells. It is also known that EVs from human UC-MSC inhibit the development of bladder carcinoma cells [91] and exosomal miRNA from AD-MSC suppress the proliferation of ovarian cancer cells [92]. All if this is very relevant if we take into account that cancer cells internalize a greater amount of EVs than normal cells [93,94].

### 4.5. Antifibrotic Effects

Fibrotic effects consist of an accumulation of ECM proteins, such as collagen I, collagen III, and fibronectin. This condition is consequence of subjacent mechanisms such as inflammation, oxidative stress, and aging. There are data from in vivo studies showing the antifibrotic effect of Warthon jelly MSC against skeletal muscle fibrosis [95] or renal or hepatic fibrosis [25,96], via the secretion of MMP-1, VEGF, and HGF, respectively.

### 4.6. Antimicrobial Effects

It is important to consider that, apart from the vulnerability to infections due to mechanisms associated with senescence, the role of the microbiome at any part of the organism may contribute to inflamm-aging. [97,98]. With aging, there is a disbalance between the commensal good microbes and the bad microbes, especially in the gut [99,100,101]. In this context, the possible antimicrobial homeostatic role of MSC may be relevant. This is due to their expression of interferon (IFN) and their stimulated genes (ISGs), which include CCL2, IFI6, PMAIP1, p21/CDKN1A, ISG15, and SAT1. In addition, members of the ISG family can prevent viral infections before viruses cross the lipid bilayer. Consequently, MSC are usually resistant to viral infection [102,103].

MSC might also act directly through the secretion of antimicrobial peptides. These small effector molecules, with 10–150 amino acids such as cathelicidin, defensins, cystatin C, elafin, and lipocalin 2 [104,105], may disrupt the microbial membrane integrity and inhibit their protein, DNA, or RNA synthesis [106]. Nevertheless, the main mechanism of action is by a cleavage product of the cathelicidin, which has a broad range of antibacterial [107,108,109], antifungal [110], and antiviral [111] activities. IL-10, IDO, IL-17, and prostaglandin E2 (PGE2) are other proteins secreted by MSC that have antimicrobial activity [112,113].

## 5. MSC and Aging

**Figure 2:**
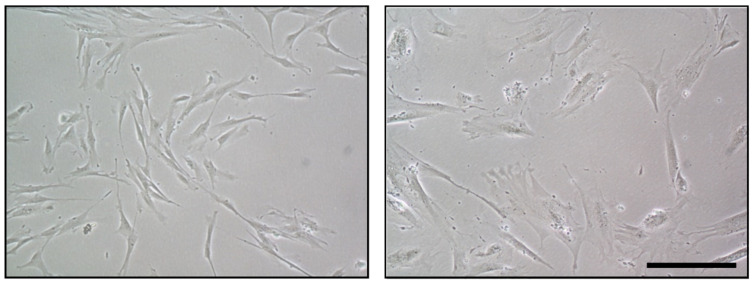
Morphological differences between proliferative (**left**) and senescent cells (**right**) (10×), human mesenchymal cervical stem cells (hUCESC). Scale bar: 50 µm.

Replicative senescence may be activated in MSC and undergo many cell divisions. This may be, in part, due to telomere shortening, which leads them to replicative senescence [114]. In addition, there are other mechanisms that lead MSC to senescence, such as the above-mentioned oncogene-induced senescence [115,116,117,118], stress-induced premature senescence [119,120], epigenic alterations [121], autophagy [122], and mitochondrial dysfunction [123]. All of these pro-aging mechanisms implicate both morphological and functional changes in senescent MSC. Morphological changes include enlarged and more granular morphology and nuclei with small and condensed spots of heterochromatin structure [124] (Figure 2). They also have a decrease in cell colony numbers (CFU) [15] and an increase in the expression of SA-β-gal [125]. In addition, they have a prolonged G1/G0 phase of the cell cycle and a significantly decreased S phase [126]. On the other hand, senescent MSC tend to change their differentiation potentials. Specifically, aged MSC exhibit a reduced osteogenic ability, while an increased adipogenic differentiation ability [127] contributes to decreased bone formation [128] and osteoporosis [129]. Senescent MSC also have a low capacity to recruit macrophages and fail to polarize them toward the anti-inflammatory M2 phenotype [130].

Interestingly, senescent MSC affect their neighboring cells by secreting the above-mentioned SASP factors [131], which are also responsible for inflamm-aging [132]. Senescent MSC also change the composition of their exosomes (for review, see [133,134]). Specially circulating exosomes from elderly donors reduce the osteogenic potential of young MSC, which may be by secreted microvesicular miR-31 (for review, see [135]).

Several studies showed that aging is generally associated with increased EV production by senescence cells [136,137,138]. It has been reported that EV secretion by senescent cells is at least in part dependent on p53 and its downstream target gene tumor suppression-activated pathway 6 (TSAP6) [139]. There are two possible benefits of this physiological finding. On the one hand, the enhanced secretion of EVs from senescent cells may facilitate cell survival by the removal of toxic molecules (for review, see [140]). On the other hand, the release of EVs by senescent cells may be a protective mechanism for signaling the distress, by which neighboring cells may respond more adequately to stress. However, it is more possible that those EVs produced by senescent cells integrate the collation of SASPs that, as a whole, will contribute to strong prosenescent signals.

### 5.1. Functional Alterations of MSC in Aging and Related Diseases

Functional alterations of MSC in aged-related diseases have been reported: for example, in osteoarthritis (OA), idiopathic pulmonary fibrosis (IPF), or cardiovascular diseases.

Diseases of the musculoskeletal system are classically age-associated and are gaining relevance because they are assosciated with disability; they are related with an increased risk of falls, depression, and higher mortality [141]. It has been found that there is a higher number of MSC in the OA cartilage than in the normal cartilage [142,143], but they show alterations [144]. Senescent MSC of the human OA cartilage, when injected intra-articularly, were sufficient to induce cartilage degeneration. In addition, senescent MSC of human OA cartilage are prone to osteogenesis rather than chondrogenesis [145].

IPF is a disease leading to irreversible loss of lung function [146]. There are data indicating a link between senescent MSC and the onset of the IPF [147]. In fact, when human lung fibroblasts were cultured with IPF MSC, an increased expression of senescence markers, such as SA-β-gal, p16INK4A, and p53, was found.

A group of self-renewal multipotent cardiac stem and progenitor cells in human heart has been identified [148], the functions of which are decreased by aging, leading to cardiovascular disease [149]. Analysis of these cells isolated from patients with cardiovascular diseases revealed how they have a reduced capability for self-renewal and differentiation, which may be due to the increased expression of SASPs, including MMP-3, GM-CSF, PAI1, IL-1β, IL-6, and IL-8 [150].

## 6. MSC as Basis of Anti-Aging Strategies

Aging frailty is a complex geriatric syndrome caused by the disruption of physiological homeostasis. Frailty can be identified by at least three of these components: slow walking speed and low physical performance, self-reported exhaustion, weakness and unintentional weight loss [151]. Notably, the brain (brain atrophy, loss of neurons, and synapse connections) [152], cardiovascular system (myocardial infarction, atrial fibrillation, and chronic heart failure) [153,154], skeletal muscle (progressive loss of muscle mass and strength) [155,156], and endocrine system (imbalance between the anabolic and catabolic [157,158,159]) are intrinsically interrelated to frailty (reviewed by [160,161]).

The management of geriatric patients consists of the implementation of calorie restriction, exercise regimes, and hormonal supplementations [37,61,162]. Diets high in n-3 polyunsaturated fatty acids and vitamin D in reduced levels of inflammatory cytokines reduce the mortality of the inflammatory diseases [163,164]. Although including exercise and/or nutrition and/or cognitive training have good results [165], these personalized approaches have failed to produce consistent results for frailty [165]. Thus, new strategies are necessary that allow us to delay the onset of frailty.

Moreover, we have mentioned above evidence indicating the recognized role of MSC in tissue homeostasis; strategies based on their positive biological effects or their secretome-derived products may be an alternative for aging frailty. Therefore, the mechanisms associated to these biological effects might face to relive several frailty symptoms, such as unintentional weight, muscle loss and weakness, a feeling of fatigue, and low levels of physical activity [160]. In this sense, Lui et al. proposed the “3Rs”: remove, rejuvenate, or replace (3Rs) the senescent MSC [166]. In addition, we propose here the new concept of “Exogenous Restitution of Intercellular Signalling of Stem Cells” (ERISSC).

### 6.1. Senolytics: Elimination of Senescent Cells

Experimental studies reported that senolytic drugs eliminated senescent cells in mice and alleviated age-related diseases, such as muscle loss, pulmonary fibrosis, metabolic syndrome, osteoporosis, cardiac dysfunction, vascular dysfunction, diabetes, and dementia [167]. Several BCL-2 family inhibitors (dasatinib, quercetin, or ravitoclax) have been used by this proposal. It was basic that the BCL-2 gene family plays a central role in the regulation of programmed cell death [168,169,170]. In fact, patients with IPF, treated with the senolytics dasatinib and quercetin, showed significantly improved physical performance in a first human clinical trial [171]. Other drugs inhibiting BCL-2 family members, such as A1331852 and A1155463, have also been promising [172].

### 6.2. Rejuvenation of MSC

Approaches to the rejuvenation of MSC may be based on antioxidant drugs, autophagy regulation, microRNA treatment, and preconditioning modification and genetic modification [173].

MSC senescence can be reversed by reducing RO production with antioxidants such as ascorbic acid, lactoferrin, N-acetyl-L-cysteine, or Cirsium setidens [174,175,176,177].

Regulating the autophagy level is also a strategy to rejuvenate senescent MSC. Treatment with rapamycin, an autophagy inhibitor, remarkably downregulated SASP in senescent MSC [178]. In addition, it was shown that inhibition of mTORC1 with AICAR and NAM boosts autophagy and postpones senescence-associated changes [179]. Melatonin can also reverse MSC senescence through upregulation of HSPA1L, a heat shock protein, which increases the antioxidant enzyme activity in mitochondria from senescent MSC [180].

Genetic engineering is another strategy used to slow MSC aging, for which several molecules have been identified as potential targets (reviewed by [181]). Thus, for example, it has been reported that ectopic expression of telomerase reverses transcriptase in MSC-abolished senescence [182]. The introduction of Erb-B2 receptor tyrosine kinase 4 (ERBB4) in aged MSC rescues the senescence phenotype and conferred resistance to oxidative stress [183]. Overexpression of macrophage migration inhibitory factor (MIF) rejuvenates aged MSC by activating autophagy [184]. On the other hand, it has been shown in in vitro studies that microRNAs can regulate MSC senescence (reviewed by [125]). Thus, for example, MiR-1292 regulates senescence and osteogenesis through the Wnt/β-catenin signaling pathway [185]. Further in vivo studies targeting several senescent-associated microRNAs in MSC are necessary to achieve an effective effect.

### 6.3. Replacing MSC

Considering that the chronological age of a donor strongly conditions the quality and lifespan of MSC [186,187], it would be reasonable to use their allogenic younger MSC due to their higher proliferative capacity and differentiation potential compared with those from aged donors [188]. In fact, there is recent evidence supporting this strategy. It has been shown that allogeneic MSC transplantation reduced the serum TNF-α in older patients [189]. This is an interesting finding because serum TNF-α is considered an inflammatory marker linked with age-related chronic diseases [190]. In a model of traumatic brain injury, it was found that functional recovery was remarkable after the transplantation of MSC from younger rats compared with those from older rats, which demonstrates that the neuroprotective properties of MSC are age-dependent [191]. More specifically, a recent study in patients with dilated cardiomyopathy demonstrated that with the myocardial injection of young allogenic, but not aged autologous, MSC improved their cardiac function [190].

It also worth considering that the transplantation of MSC can be utilized as treatment of several diseases, such as traumatic brain injury [192], spinal cord injury [193], cardiovascular diseases [194], stroke [195], and liver diseases [196]. Taken together, this led us to consider that the transplantation of MSC may serve as a therapeutic strategy in aging frailty [37,197]. Two clinical trials—phase I and phase II—were conducted in aged patients by administering different doses of allogeneic BM-derived MSC. In the first clinical trial [198], five patients in each group intravenously received 20 million, 100 million, and 200 million, respectively. All the patients had an increased 6 min walk distance at 3 months and 6 months, and their serum levels of TNF-α decreased. In a consecutive clinical trial [199], in which 30 patients with aging frailty were randomized into 100 million, 200 million was found to be safe and immunologic improvement detected in the two treatment groups. In addition, these two trials confirmed that 100 million cells were better than 200 million cells. One possible explanation may to be related to the potentially deleterious effects of higher doses on cell retention or cell survival. On the basis of these preliminary clinical studies, and the impending phase III clinical trials, we may have to consider that MSC transplantation might be a possible innovative treatment of frailty. However, caution is still needed when considering the limitations of cells therapies, as shown in the following.

### 6.4. Therapy Based on MSC Secretome-Derived Products: A New Proposal for Aging Frailty Treatment

**Figure 3:**
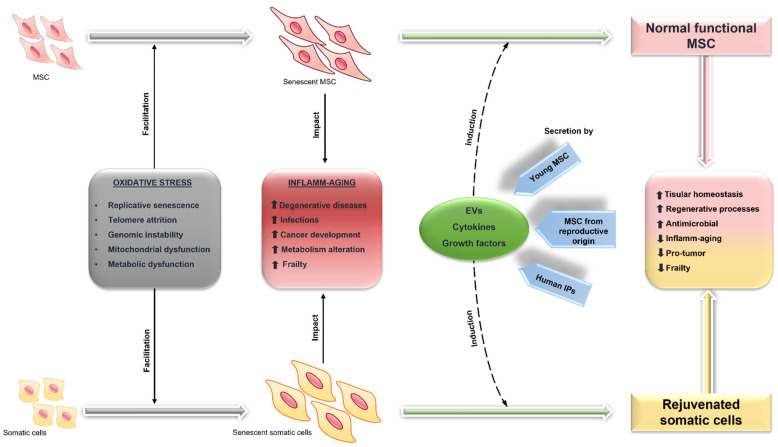
Physiopathological mechanisms associated with the senescence of MSC (mesenchymal stem cells) and somatic cells, as well as the “Exogenous Restitution of Intercellular Signalling of Stem Cells” (ERISSC) strategy.

Based on the hypothesis that MSC contribute to the maintenance of tissue homeostasis by the secretion of a “galaxy” of intercellular signals [200], we can propose the new concept of “Exogenous Restitution of MSC Intercellular Signalling of Stem Cells” (ERISSC) (Figure 3). This close-up may be possible by using the strategy of MSC secretory-derived products, which may be obtained through in vitro cultures of these multifunctional cells.

The initial idea, based on their ability to home injury sites though cell differentiation and so contributing to regeneration, was abandoned due to the implantation time of MSC, which is usually too short to have an effective impact [201,202]. In fact, it is now known that the survival of MSC is less than 1% one week after systemic administration [67,203,204], and their contribution to the tissular regeneration is generally minimal [205]. Nonetheless, there are many biological effects of promoting cell-to-cell interactions and cellular proliferation [206,207], and the more recent data indicate that the majority effects of MSC are due to the secretion of paracrine factors, such as growth factors, cytokines, and EVs, which overall display regenerative, anti-inflammatory, angiogenic, antiapoptotic, or antioxidative stress properties. MSC secretome may represent a new medical biotechnology [208]. This makes MSC secretome a potentially ideal strategy to restore the physiological tissular homeostasis after virulent senescence progressive development.

Preliminary reports show the efficacy of secretome-derived products from MSC, such as CM or EVs, in experimental models related to aging. It was reported that CM from human fetal MSC significantly reduced SA-βGal expression, ameliorated replicative senescence of adult MSC, and enhanced their cell proliferation and differentiation potential [209]. A similar approach demonstrated that CM from MSC regulated senescence features in IL1β-treated OA chondrocytes, reducd the number of actin stress fibers and accumulation of γH2AX foci, and decreased the oxidative stress [210]. In a very recent study, the fetal dermal MSC-CM improved the anti-aging effects against adult dermal fibroblasts induced in an experimental model [211].

With regard to MSC-EVs, there are data showing that these vesicles from healthy donors downregulate SA-βGal activity and γH2AX in osteoblasts isolated from OA patients [212], as well as reducing the oxidative stress and the proinflammatory cytokines PGE2 and IL6. EVs from young MSC also reduce senescent features of MSC after passages 10–14 [213]. In addition, it was shown that the administration of EVs from young mice reduces senescence in mice [214]. In this sense, it was recently reported that EVs derived from young MSC rejuvenate senescent endothelial progenitor cells (EPCs) [215]. This is a relevant finding when one considers that, following ischemic events, EPCs are mobilized from the bone marrow and migrate to sites of vascular injury, promoting angiogenesis [216]. However, aging-associated EPC senescence increases the risk of ischemic events [217]. EVs from young MSC were shown to rejuvenate hematopoietic stem cells by transferring autophagy- and lineage commitment-related mRNAs [218] and also have a protective effect in liposaccharide-induced acute lung injury [219].

Nevertheless, the application of this new concept of the extracellular restitution of intercellular signals from stem cells (ERISSC), based on cell-free therapies, will create a great challenge.

## 7. The Challenge to Implement a Therapy Based on ERRISC for Aging Frailty Treatment

To face this ambitious challenge, we have to clarify key strategy aspects, such as establishing tools that allow us a more precise diagnosis of the aging frailty in order to identify the therapeutic requirements adapted to each case, to identify the ideal type of MSC in the contest of functional heterogeneity of these cellular populations, to optimize the mass production and standardization of the primary materials (cells) and their secretome-derived products, to establish the appropriate methods to validate the anti-aging effects, to explore the mechanisms, and to determine the most appropriate route of administration for each case.

### 7.1. More Precise Diagnosis of the Aging Frailty

In order to attain a more precise approach to the biological and functional diagnosis of the frailty that allows us to monitor the therapeutic possibilities and, thus, adapt the possible treatments, we will need more precise biological markers of this complex process. We will probably have to integrate the previously mentioned classical molecular markers of senescence, such as A-βGal or SA-α-Fuc, with new tools to monitor MSC aging, such as SiR-actin [220] and/or CyBC9 (another fluorescent probe), which contribute to evaluating the loss of membrane potential of mitochondria in senescent MSC [221]. On the other hand, senescent late-passage MSC secrete larger amounts of MSC-derived microvesicles (MSC-MVs) than those in early passages, and their RNA sequencing suggests that most genes are involved in aging-related diseases [134]. Therefore, there is a need to integrate all these senescence markers, probably by employing bioinformatics-based analyses in order to provide potential drug targets for senescence intervention.

### 7.2. The More Suitable Source of MSC

Today, there are no data suggesting the best source of MSC for clinical use in frailty. The heterogeneity between MSC according to their origin as well as the different protocols used in their culture and expansion make their comparison difficult. BM-MSC were initially widely used. However, some of their drawbacks, such as the invasive method to obtain them and their low cell yield (0.001–0.01% of bone marrow mononuclear cells), have motivated the search for other tissue sources. Although it is possible to obtain peripheral blood-derived MSC (PB-MSC) mobilized by the G-CSF, both BM-MSC and PB-MSC have a longer doubling time in vitro compared to MSC from other sources [222]. Other options are ASC-MSC, which can be obtained easily as surgical waste and lipo-aspirates at a high concentration up to 3%, or UC-MSC, which have a higher degree of multipotency than BM-MSC and ASC [62]. More recently, dental pulp-derived MSC (DPSCs) have been identified in the surgical removal of wisdom teeth. DPSCs present high proliferative [223] and differentiation capabilities; for this reason, they have been proposed in regenerative therapies [224]. Interestingly, DPSCs have not only the potential to differentiate into dentin-forming odontoblasts [225] or to induce bone regeneration [226], but also neurogenic capability, perhaps due to their neural crest origin [227]. DPSCs have been positively applied in clinical trials for the surgical treatment of intrabony periodontal defects with promising results [228], and they are currently under development as a cell-based therapy for ischemic stroke [229].

Another factor that one should keep in mind, in addition to the tissue origin source from MSC, is the heterogeneity among donors, which may be independent of the chronological age. One biological function that significantly influences biological age is reproductive function [230]. An association between shorter life spans and reproductive failure for a cohort of men has been demonstrated [231]. In this context, cardiovascular disease is rare in premenopausal women, but this process increases during menopause or in young women with premature ovarian failure [232,233]. On the other hand, humans are the one species in which a consistent gender-specific survival advantage exists. Women live longer than men. However, adult women generally appear to be in poorer health than men. This phenomenon, termed “the mortality-morbidity paradox”, is seen only in humans [234,235]. Based on all of these premises, one led us to consider the hypothesis that MSC from female active reproductive system may to a relationship with all of these favorable conditions from women and, therefore, be a good candidate to obtain secretome-derived products for regenerative strategies. In accordance with it, there is evidence indicating that the uterus from nonpregnant, premenopausal women has a surge of MSC, such as those of endometrial or cervical origins, which have a wide differentiation capability into many cellular linages, as well as a potential therapeutic effect in several pathological processes (reviewed by [236]). In addition, human mesenchymal cervical stem cells (hUCESC) secretome shows potent anti-inflammatory [237,238], regenerative [238,239,240], antimicrobial [239,241] and antitumor properties, this latter aspect being very relevant in consideration of the protumor senescent condition and the recognized current limitations of MSC cellular therapies, such as protumor risk [242].

### 7.3. The Need for Mass Production of MSC

Due to the minute amount of MSC in organs and tissues, the in vitro expansion of MSC is necessary for clinical applications [16,242]. However, after prolonged expansion in cultures, senescence-induced alterations in their function may be experimented in MSC [243]. Thus, once the ideal MSC have been chosen, their expansion in cell cultures is necessary.

It was estimated that the possible number of passages that can be achieved by MSC in cultures is from 30 to 40 [244,245]. The differentiation potential of MSC decreases after extended passages [246]. Therefore, strategies that allow obtaining a large number of MSC while retaining their stemness are appropriate. Another possibility is to induce pluripotent stem cell (iPSC)-derived MSC (iMSC), which can be passaged many times without showing signs of senescence [247]. However, this process requires molecular manipulation and has the risk of tumorigenicity after in vivo transplantation of MSC [248].

On the other hand, identifying an effective large-scale expansion technique is essential to obtain the necessary high number of cells without sacrificing the cell quality. The classic T-flasks production system is only profitable to obtain MSC for therapeutic purposes for few patients. For larger clinical trials, the mandatory resources for cell culture would become insupportable [249]. Bioprocessing strategies for large-scale production are multilayered flask, spinner flask, roller bottle, or bioreactor. In particular, the expansion of MSC in bioreactors permits large-scale expansion in a cost-effective manner [250,251]. In addition, it permits greater traceability, elimination of contamination, inappropriate fluctuations in pH, oxygen concentration or nutrients.

Stirred tank reactors are the most widely used devices for large-scale MSC expansion [252]. Among the technological keys in these cell culture expansion systems are the microcarriers and hydrodynamic parameters. Microcarriers are made of diverse materials with different surface properties [62], which provide a high surface-to-volume ratio for high-density cell cultures, allowing the growth of cultures in 3D [253,254,255]. Hydrodynamic parameters include aeration and agitation. Aeration is required to culture cells with the optimal supply oxygen. However, in bioreactors, aeration must be actively aerated by, e.g., bubbling the gas into the liquid, which generates strong forces that can damage cells [256]. Therefore, agitation in bioreactors is necessary to disperse gas.

Culture modifications to improve the therapeutic interest of MSC comprise oxygen and pH. The oxygen saturation in standard T-flasks (21% O_2_) is quite distant from nature (5–7% O_2_). There are data indicating that maintaining cells at a low pH [257] or a low O_2_ tension [258,259,260,261] can improve the release and capacity of exosomes.

### 7.4. Standardization, Functional Test for Anti-Aging Effects, and Mechanisms

It has been demonstrated how both molecular and functional differences among MSC are from the same origin but are produced in different laboratories [262]. These situations may occur because there is no standardized protocol for the MSC manufacturing process. Many studies reported variations in MSC functionality in accordance with both the donor-to-donor and tissue source [263,264,265,266] and also with regard to different culture or preconditioning strategies [267]. Likewise, the impact of bioprocessing parameters on MSC therapeutic potency have been reported [268,269]. For all of this, it is necessary not only to minimize donor-to-donor variability, but also to harmonize and standardize the large-scale expansion of MSC in order to reduce the bioprocessing variability before allogenic cell therapies or based on their derived-secretomes.

On the other hand, the implementation of acceptable potency assays of MSC for clinical therapies that predict their in vivo efficacy is necessary [134].

In vitro functional tests of the effects of the biological products should be performed before anti-aging treatment. On the other hand, the studies of the mechanisms of action are important aspects, which may be required by regulatory agencies. In addition, we have to consider that, traditionally, there is a low inclusion of geriatric patients in clinical trials, and most therapies were validated on experimental models using young animals [270]. Considering this, ideally experimental models that reflect exactly the same sudden biological changes undergone by the aging of the human body should be used.

### 7.5. The Selection of Administration Route

Another no less important aspect to solve is the choice of the appropriate route of administration for each case. If we take as a reference the studies based on cell therapy, local administration of MSC that targeted the injury site produced rapid results, but there also is a risk of cell death and bleeding [90]. When we compare the different methods of systemic administration of MSC, we observe in some cases different effects. MSC administered through the intravascular (IV) route tend to become entrapped in the lungs [90,271]. The preference for intraperitoneal (IP) over IV as to avoid the risk of pulmonary embolization has been reported (Roux et al. [272]). In addition, IP infusion of MSC produced better homing and inflammation suppression than IV (Castelo-Branco et al. [273]). On the contrary, the IV administration of MSC was more effective than the IP method in the treatment of colitis (Gonçalves et al. [274]). IM injection of MSC, which is less invasive than IV, produces the longest cell retention (more than 100 days) when compared to IV, IP, and subcutaneous (SC) (Braid et al. [271]). On the other hand, IV-infused MSC can cross the blood–brain barrier (BBB), which is essential for efficacy [275,276,277]. Administration of MSC via intracerebral and intrathecal routes also showed positive results in experimental studies [278,279].

In this scenario of the different modes of administration of products in the field of regenerative medicine, the use of derivatives of the secretome of MSC opens up new, interesting expectations. Regarding topical applications, there is the possibility of using tailored hydrogels of these new products, as we recently described for the treatment of experimental colitis using a mucoadhesive hydrogel containing CM-hUCESC [238]. Along the same lines, the possible topical or local application (for example, intra-articular) of products derived from the secretome of MSC combined with materials that, based on nanotechnology, allow their slow and sustained release can be very interesting. Regarding systemic administration, we can also take advantage of the strategic interest of exosomes for therapies. They are smaller and less immunogenic than their progenitor cells [280]. Their storage is easier, they have a longer half-life in the blood stream [281], tropism towards inflamed tissues and tumors, and the ability to cross the blood–brain barrier [282,283]. Regarding this latter aspect, interestingly, it was shown that secretome derived from MSC administered intranasally re-established mouse memory in an experimental model of Alzheimer’s disease [284].

## 8. Discussion and Conclusions

Aging and frailty are complex processes implicating multifactorial mechanisms, such as replicative senescence, oxidative stress, mitochondrial dysfunction, or autophagy disorder. All of these mechanisms drive dramatic changes in the tissue environment, such as SASP and inflamm-aging. Although aging and aging-related pathophysiological changes are inevitable, and for which there are no treatments, society demands strategies that promote this biological process to be at least healthier, mainly with regard to delaying as much as possible or avoiding the state of fragility. However, at present it has not been possible to identify the ideal strategies for this purpose. The implementations of personalized approaches, such as dietetics, exercise regimens, or hormonal supplementations, have failed to produce consistent results [165]. Therefore, it is necessary to find strategic alternatives different from the classic ones.

Considering that the biological mechanisms associated with aging translate into a complex alteration of tissue homeostasis, MSC could be a good anti-aging strategy. This is because MSC influence the intercellular communication by controlling several aged-related processes and associated diseases, such as anti-inflammatory, regenerative, and antioxidative stress, and antifibrotic, antitumor, or antimicrobial effects. In fact, it has been demonstrated that an alteration in the functionality of MSC is related to early aging syndromes and age-related diseases [142,143,144,145,146,147,148,149,150]. These relatively new concepts have led to the attractive 3R strategy: remove senescent MSC, rejuvenation, or reprogramming [166]. However, these possibilities show important limitations, starting with the fact that MSC are not immune to senescence.

Removing MSC in an aging organism may have unknown consequences. It was suggested that the abrupt removal of senescent cells can result in the loss of normal cellular reprogramming, wound healing, and tissue regeneration [285,286,287]. In addition, long-term use of senolytic drugs might have side effects, such as severe thrombocytopenic and neutropenic effects [288,289].

Rejuvenation of MSC by systemic administration of high doses of ant-oxidant agents can produce DNA damage and induce premature senescence [290]. Thus, the use of this strategy needs to be carefully evaluated. In addition, genetic engineering to slow MSC aging can increase the risk of malignant transformation.

Restitution aging MSC by younger MSC has a priori all the drawbacks associated with cell therapy, but especially the recognized low rate of implantation of transplanted MSC [67,203,204].

Based on all of these limitations of potential strategies based on MSC, we consider that “Exogenous Restitution of Intercellular Signalling of Stem Cells” (ERISSC) may be an attractive option. This proposal seems reasonable if we consider that MSC seem to exert their positive effect on tissue homeostasis through a paracrine mechanism by producing a cocktail of proteins and extracellular vesicles that impact positively with host cells [72]. The use of this product as a therapeutic strategy can help avoid the different drawbacks of direct cell therapy with MSC, among them, immunological incompatibility, the formation of emboli or transmissible infections, and tumorigenicity. In addition, unlike cell therapies, the use of secretome has additional advantages such as better evaluation of the dose and potency and safety, and it can be stored without the application of toxic cryopreservative agents. Thus, the use of products derived from the secretome is more practical for clinical use and could be prepared in advance for treatments when necessary [291].

Another important advantage of cell-free therapy based on secretome, as opposed to cell therapy, is to avoid the absence of a biological effects of MSC in the progressively most frequent older patient population compared with young people. This circumstance has been attributed to a tissue microenvironment related to senescence, which includes the above-mentioned production of bioactive factors such as inflammatory cytokines, chemokines, growth factors, and proteases. All of them, collectively labeled the SASP, contribute to a particularly hostile environment for MSC viability and functionality, which leads to an unsatisfactory effect of this type of cell therapy [125]. It is, therefore, indispensable to find new future strategies for this age group that demand so many new therapies.

For ERISSC, we propose that several challenges should be faced. In the first place, strategies for monitoring senescence and identifying the possible intervention opportunities are necessary. In addition, to obtain an optimal MSC secretome, their proper standardization and mass production are required, as well as the use of functional assays to test the obtained biological products and appropriate experimental models to explore the mechanisms. With regard to these latter aspects, new emerging tools such as human organoid cultures could be the model for research dedicated to aging in some therapeutic fields [292]. Fortunately, there are human MSC of anatomical origin and attractive biological potential for these purposes, such as DPSCs or hUCESC. In addition, the appropriate technology is being developed for MSC expansion and mass production of their secrets by using bioreactors. There are also new tools to establish the more adequate mode of administration for MSC secretome-derived products, such as the use of tailored hydrogels for topical applications [238] or MSC-derived exosomes for systemic therapies [282,283]. All of these advantages permit us to challenge this. The only failure would be not trying.

## Data Availability

Not applicable.
